# *In vitro* inhibitory effects of cepharanthine on human liver cytochrome P450 enzymes

**DOI:** 10.1080/13880209.2020.1741650

**Published:** 2020-03-28

**Authors:** Xunge Zhang, Ping Feng, Xinfu Gao, Bin Wang, Chunxia Gou, Ruimin Bian

**Affiliations:** Department of Pharmacy, Binzhou Medical University Hospital, Binzhou, PR China

**Keywords:** CYP3A4, CYP2E1, CYP2C9, drug–drug interaction

## Abstract

**Context:**

Cepharanthine (CEP) extracted from the roots of *Stephania cepharantha* Hayata (Menispermaceae), has a range of therapeutic potential in clinical conditions. Whether it affects the activity of human liver cytochrome P450 (CYP) enzymes remains unclear.

**Materials and methods:**

The effects of CEP (100 μM) on eight human liver CYP isoforms (i.e., 1A2, 3A4, 2A6, 2E1, 2D6, 2C9, 2C19 and 2C8) were investigated *in vitro* using human liver microsomes (HLMs) with specific probe actions and probe substrates. In addition, the enzyme kinetic parameters were calculated.

**Results:**

The results showed that the activity of CYP3A4, CYP2E1 and CYP2C9 was inhibited by CEP, with IC_50_ values of 16.29, 25.62 and 24.57 μM, respectively, but other CYP isoforms were not affected. Enzyme kinetic studies showed that CEP was not only a non-competitive inhibitor of CYP3A4 but also a competitive inhibitor of CYP2E1 and CYP2C9, with *Ki* values of 8.12, 11.78 and 13.06 μM, respectively. Additionally, CEP is a time-dependent inhibitor for CYP3A4 with *K_I_*/*K_inact_* value of 10.84/0.058 min/μM.

**Discussion and conclusions:**

The *in vitro* studies of CEP with CYP isoforms indicate that CEP has the potential to cause pharmacokinetic drug interactions with other co-administered drugs metabolized by CYP3A4, CYP2E1 and CYP2C9. Further clinical studies are needed to evaluate the significance of this interaction.

## Introduction

Cepharanthine (CEP) is a biscoclaurine alkaloid, extracted from the roots of *Stephania cepharantha* Hayata (Menispermaceae), known to have anti-inflammatory and immunomodulatory activities (Fujii et al. 1988). It has been reported that CEP has a range of therapeutic potential in clinical conditions (Shinobu and Jianghong 2007; Rogosnitzky and Danks 2011). Conditions that have been reported to benefit from CEP therapy including radiation-induced leukopoenia (Suzuki et al. 1992), idiopathic thrombocytopenic purpura (Kobayashi et al. 1992), alopecia areata and alopecia pityrodes (Nomoto et al. 2004). Moreover, it is known to have antitumor activity, antitumor invasion and pro-apoptotic action in many cancer cells (Bun et al. 2009). CEP mitigates lung injury induced by bilateral lower limb I/R in rats (Kao et al. 2015), and it might have a potential innovative antiplasmodial mechanism of action (Desgrouas et al. 2014). With the development of the treatment of tumours, CEP has become more and more popular.

Cytochrome P450 (CYP) enzymes are membrane-bound hemoproteins, that play a vital role in the biotransformation of xenobiotics, including drugs, environmental pollutants, carcinogens and endogenous substrates (Wrighton and Stevens 1992; Yan and Caldwell 2001). CYPs have their own specific nomenclature and classification. Among its family, CYP1A, CYP2C, CYP2D, CYP3A and CYP2E are major CYP enzymes in drug metabolism (Li 2001). A number of factors affect the expression and function of CYPs. The induction or inhibition of CYPs is a major mechanism that underlies drug–drug interactions (Manikandan and Nagini 2018). For example, dihydromyricetin, berberine and pristimerin have inhibitory effects on the activity of CYPs (Chang et al. 2015; Liu et al. 2017; Hao et al. 2018). Glycyrrhizic acid, verapamil and grapefruit juice may affect the pharmacokinetics of other drugs through inhibiting CYPs (Huang et al. 2018; Jia et al. 2018; Zhao et al. 2018).

CEP has been widely used in clinic; however, the effect of CEP on the activity of CYP enzymes was unclear. In this study, the effect of CEP on eight major CYP isoforms in human liver microsomes (HLMs) was investigated. *In vitro*, phenacetin (CYP1A2), testosterone (CYP3A4), coumarin (CYP2A6), chlorzoxazone (CYP2E1), dextromethorphan (CYP2D6), diclofenac (CYP2C9), S-mephenytoin (CYP2C19), and paclitaxel (CYP2C8) were used as probe substrates to determine the effects of CEP on eight CYP enzymes. In addition, enzyme kinetic studies were conducted to determine the inhibition mode of CEP on CYP enzymes.

## Materials and methods

The effects of CEP on the activity of CYP enzymes were investigated in HLMs. Through comparing the probe actions in the presence or absence of CEP, the activity of eight CYPs enzymes was obtained. The enzyme kinetic parameters were also calculated to quantify the effect of CEP. With the help of HPLC, the probe substrates were analysed.

### Chemicals

**Figure 1.**
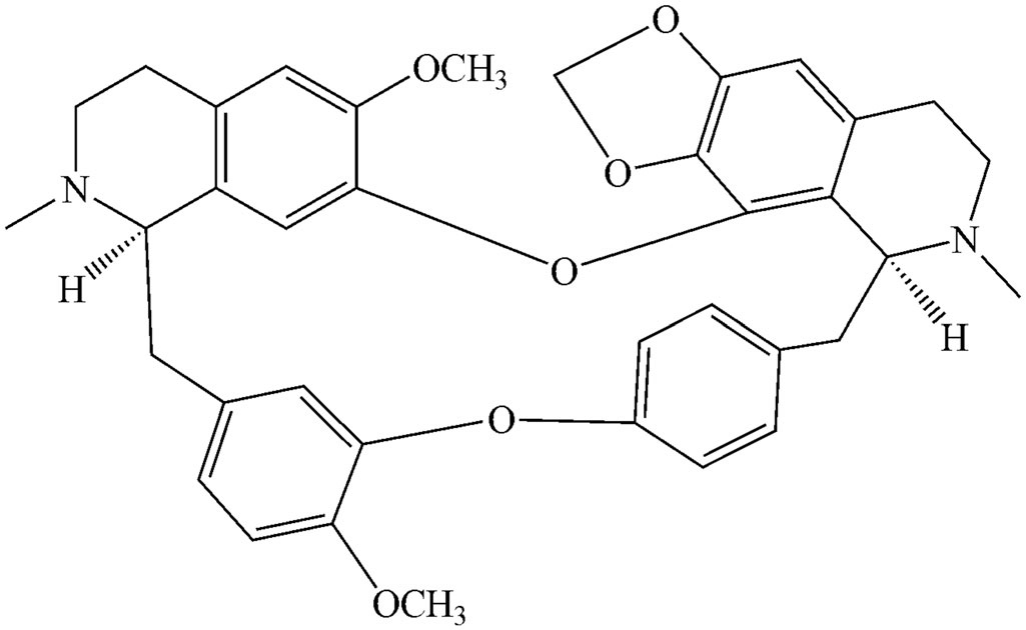
The chemical structure of CEP.

CEP (≥98%) and testosterone (≥98%) were obtained from the National Institute for the Control of Pharmaceutical and Biological Products (Beijing, China). The chemical structure of CEP is shown in Figure 1. D-Glucose-6-phosphate, glucose-6-phosphate dehydrogenase, corticosterone (≥98%), NADP^+^, phenacetin (≥98%), acetaminophen (≥98%), 4-hydroxymephenytoin (≥98%), 7-hydroxycoumarin (≥98%), 4′-hydroxydiclofenac (≥98%), sulfaphenazole (≥98%), quinidine (≥98%), tranylcypromine (≥98%), chlorzoxazone (≥98%), 6-hydroxychlorzoxazone (≥98%), paclitaxel (≥98%), 6β-hydroxytestosterone (≥98%), clomethiazole (≥98%) and furafylline (≥98%) were obtained from Sigma Chemical Co. Montelukast (≥98%) was obtained from Beijing Aleznova Pharmaceutical (Beijing, China). Coumarin (≥98%), diclofenac (≥98%), dextromethorphan (≥98%) and ketoconazole (≥98%) were purchased from ICN Biomedicals (Orange County, California). Pooled HLMs were purchased from BD Biosciences Discovery Labware (BD Biosciences, Franklin Lakes, NJ). All other reagents and solvents were of analytical reagent grade.

### Assay with human liver microsomes

As shown in Table 1, to investigate the inhibitory effects of CEP on different CYP isoforms in HLM, the following probe reactions were used, according to previously described method (Zhang et al. 2007; Qi et al. 2013; Zhang et al. 2017); phenacetin *O*-deethylation for CYP1A2, testosterone 6β-hydroxylation for CYP3A4, coumarin 7-hydroxylation for CYP2A6, chlorzoxazone 6-hydroxylation for CYP2E1, dextromethorphan *O*-demethylation for CYP2D6, diclofenac 4′-hydroxylation for CYP2C9, S-mephenytoin 4-hydroxylation for CYP2C19 and paclitaxel 6α-hydroxylation for CYP2C8. Marker reactions of eight CYP isoforms were examined, and apparent Km values were estimated to characterize the pooled HLMs. All incubations were performed in triplicate, and the mean values were utilized. The typical incubation systems contained 100 mM potassium phosphate buffer (pH 7.4), NADPH generating system (1 mM NADP^+^, 10 mM glucose-6-phosphate, 1 U/mL of glucose-6-phosphate dehydrogenase and 4 mM MgCl_2_), the appropriate concentration of HLMs, a corresponding probe substrate and hispidulin (or positive inhibitor for different probe reactions) in a final volume of 200 μL.

**Table 1.** Isoforms tested, marker reactions, incubation conditions and K_m_ used in the inhibition study.

CYPs	Marker reactions	Substrate concentration (μM)	Protein concentration (mg/mL)	Incubation time (min)	Estimated K_m_ (μM)
1A2	phenacetin *O*-deethylation	40	0.2	30	48
3A4	testosterone 6β-hydroxylation	50	0.5	10	53
2A6	coumarin 7-hydroxylation	1.0	0.1	10	1.5
2E1	chlorzoxazone 6-hydroxylation	120	0.4	30	126
2D6	dextromethorphan *O*-demethylation	25	0.25	20	4.8
2C9	diclofenac 4′-hydroxylation	10	0.3	10	13
2C19	*S*-Mephenytoin 4-hydroxylation	100	0.2	40	105
2C8	paclitaxel 6α-hydroxylation	10	0.5	30	16

The concentration of CEP was 100 μM, and the positive inhibitor concentrations were as follows: 10 μM furafylline for CYP1A2, 1 μM ketoconazole for CYP3A4, 10 μM tranylcypromine for CYP2A6, 50 μM clomethiazole for CYP2E1, 10 μM quinidine for CYP2D6, 10 μM sulfaphenazole for CYP2C9, 50 μM tranylcypromine for CYP2C19 and 5 μM montelukast for CYP2C8. Probe substrates, positive inhibitors (except for dextromethorphan and quinidine which were dissolved in water) and CEP were dissolved in methanol, with a final concentration of 1% (v/v), and 1% neat methanol was added to the incubations without inhibitor. The final microsomal protein concentration and incubation times for the different probe reactions are shown in Table 1. There was a 3-min preincubation period (at 37 °C) before the reaction was initiated by adding an NADPH-generating system. The reaction was terminated by adding a 100 μL acetonitrile (10% trichloroacetic acid for CYP2A6) internal standard mix, and the solution was placed on ice. The mixture was centrifuged at 12,000 rpm for 10 min, and an aliquot (50 μL) of supernatant was transferred for HPLC analysis. The instrument used in this study was Agilent 1260 series instrument with DAD and FLD detector, and the quantitative assay for the corresponding metabolites was performed as previously reported (Lang et al. 2016; Zhang et al. 2017). The quantitative analysis of metabolites can represent the activity of corresponding CYPs.

### Enzyme inhibition and kinetic studies of CEP

CEP (100 μM) was used to initially screen for its direct inhibitory effects towards different human CYP isoforms. For the CYP isoforms whose activities were strongly inhibited, secondary studies were performed to obtain the half inhibition concentration (*IC_50_*). *Ki* values were obtained by incubating various concentrations of different probe substrates (20–100 μM testosterone, 25–250 μM chlorzoxazone and 2–20 μM diclofenac) in the presence of 0–50 μM CEP.

### Time-dependent inhibition study of CEP

To determine whether CEP could inhibit the activity of CYP3A4, 2E1 and 2C9 in a time-dependent manner, CEP (20 μM) was pre-incubated with HLMs (1 mg/mL) in the presence of an NADPH-generating system for 30 min at 37 °C. After incubation, an aliquot (20 μL) was transferred to another incubation tube (final volume 200 μL) containing an NADPH-generating system and probe substrates whose final concentrations were approximate to *K_m_*. Then, further incubations were performed to measure the residual activity. After being incubated for 0, 5, 10, 15, and 30 min, the reactions were terminated by adding a 100 μL acetonitrile internal standard mix and then placed on ice; the corresponding metabolites were determined by HPLC.

To determine the *K_I_* and *k_inact_* values for the inactivation of CYP3A4, the incubations were conducted using higher probe substrate concentrations (approximately 4-fold *K_m_* values) and various concentrations of CEP (0–50 μM) after different preincubation times (0–30 min), with a two-step incubation scheme, as described above.

### Statistical analysis

The enzyme kinetic parameters for the probe reaction were estimated from the best fit line, using least-squares linear regression of the inverse substrate concentration *versus* the inverse velocity (Lineweaver-Burk plots), and the mean values were used to calculate *V_max_* and *K_m_*. Inhibition data from the experiments that were conducted using multiple compound concentrations were represented by Dixon plots, and inhibition constant (*K_i_*) values were calculated using non-linear regression according to the following equation:

$$v=\left(\frac{V_{\text{max}}S}{K_{m}\left(1+I/K_{i}\right)+S}\right)$$

where I is the concentration of the compound, *K_i_* is the inhibition constant, S is the concentration of the substrate, and *K_m_* is the substrate concentration at half the maximum velocity (*V*_max_) of the reaction. The mechanism of the inhibition was inspected using the Lineweaver–Burk plots and the enzyme inhibition models. The data comparison was performed using Student’s *t*-test and performed using IBM SPSS statistics version 20 (IBM SPSS Inc., Armonk, NY).

## Results

### Inhibition of CYP

**Figure 2.**
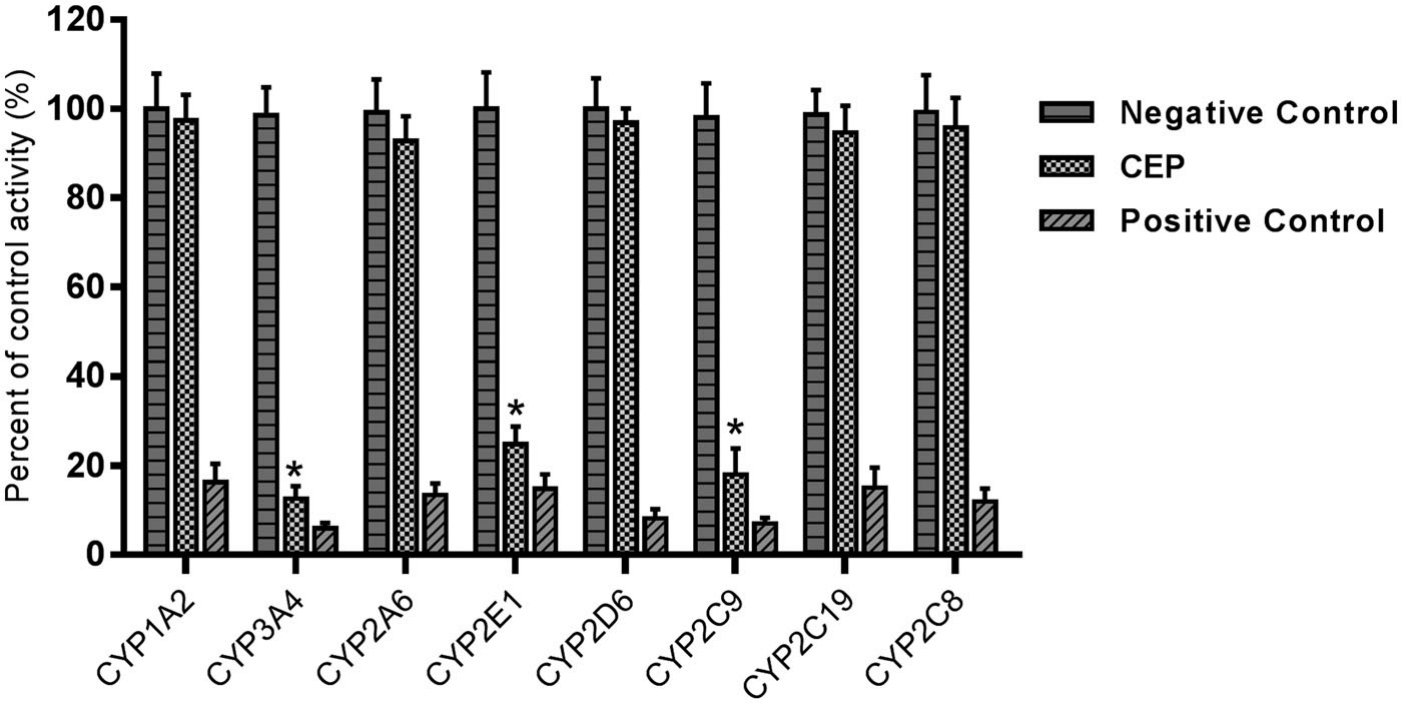
Inhibition of CEP on CYP enzymes in pooled HLMs. All data represent mean ± SD of the triplicate incubations. *p< 0.05, significantly different from the negative control. Negative control: incubation systems without CEP; CEP: incubation systems with CEP (100 μM); Positive control: incubation systems with their corresponding positive inhibitors.

The effects of CEP on the activity of CYP enzymes were investigated, through the probe reaction assays. Specific inhibitors of CYP1A2, CYP3A4, CYP2A6, CYP2E1, CYP2D6, CYP2C9, CYP2C19, and CYP2C8 were used as positive controls. As shown in Figure 2, CEP performed inhibitory effects on the activity of CYP3A4, 2E1 and 2C9, which were inhibited to 12.6%, 23.6% and 18.1% of their control activity, respectively. CYP1A2, CYP2A6, CYP2D6, CYP2C19 and CYP2C8 were not affected. Moreover, the inhibition of CYP3A4, 2E1 and 2C9 was concentration-dependent, with *IC_50_* values of 16.29, 25.62 and 24.57 μM, respectively.

**Figure 3.**
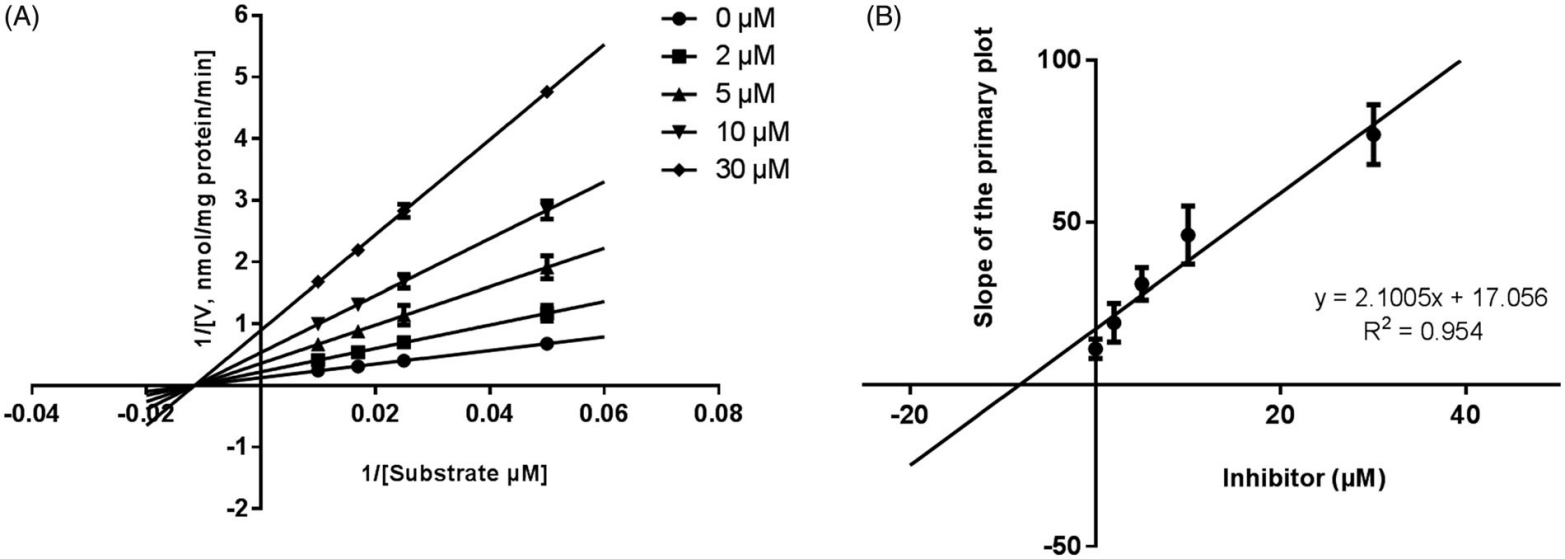
Lineweaver–Burk plots (A) and the secondary plot for *Ki* (B) of inhibition of CEP on CYP3A4 catalysed reactions (testosterone 6β-hydroxylation) in pooled HLM. Data are obtained from a 30 min incubation with testosterone (20–100 μM) in the absence or presence of CEP (0–30 μM). All data represent the mean of the incubations (performed in triplicate).

**Figure 4.**
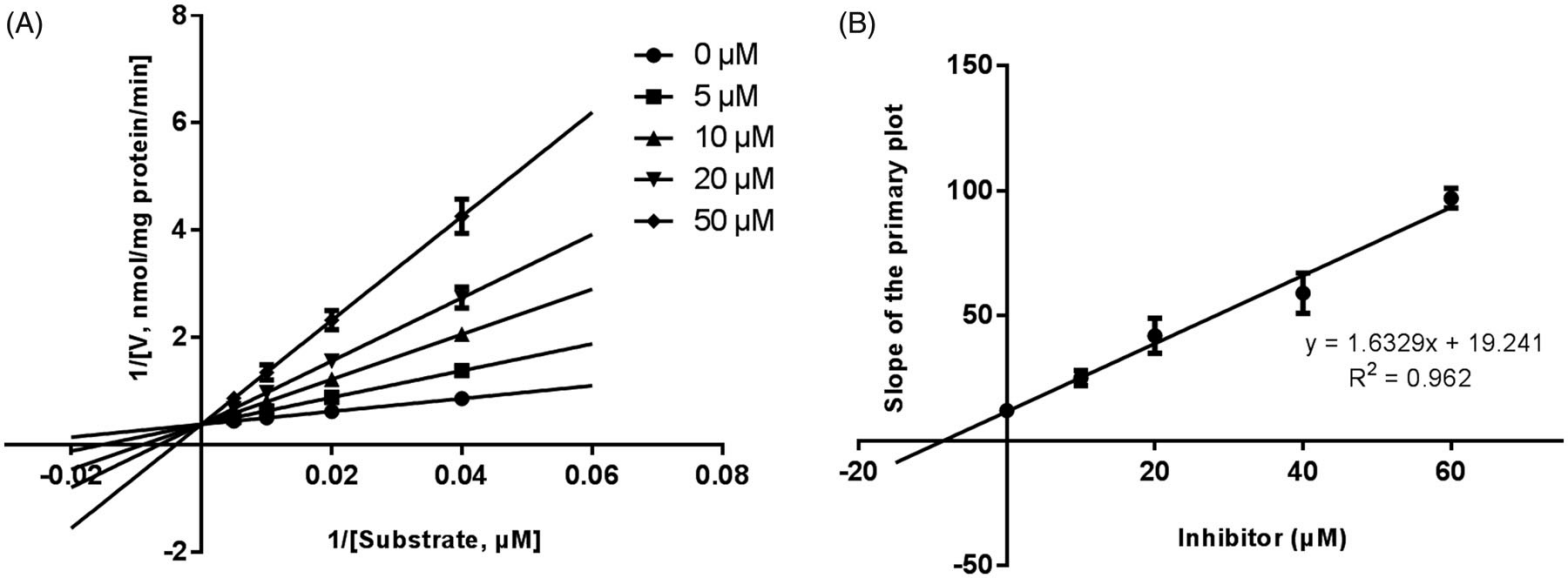
Lineweaver-Burk plots (A) and the secondary plot for *Ki* (B) of inhibition of CEP on CYP2E1 catalysed reactions (chlorzoxazone 6-hydroxylation) in pooled HLM. Data are obtained from a 30 min incubation with chlorzoxazone (25–250 μM) in the absence or presence of CEP (0–50 μM). All data represent the mean of the incubations (performed in triplicate).

**Figure 5.**
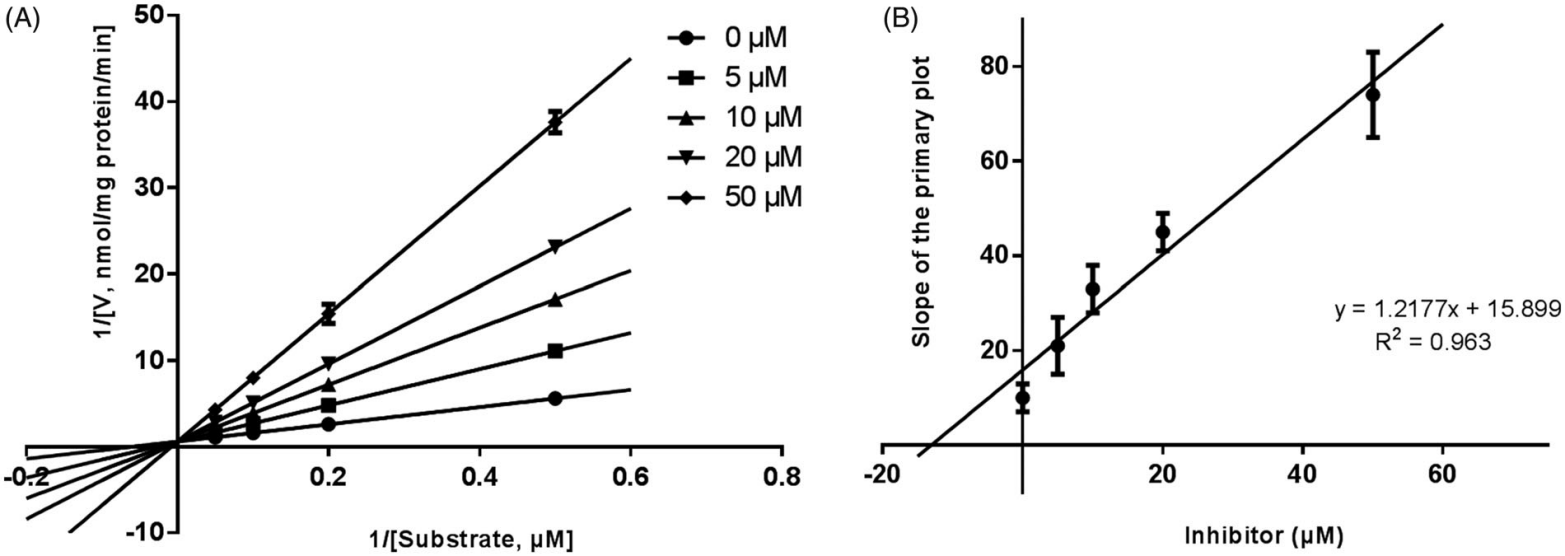
Lineweaver–Burk plots (A) and the secondary plot for *Ki* (B) of inhibition of CEP on CYP1A2 catalysed reactions (diclofenac 4′-hydroxylation) in pooled HLM. Data are obtained from a 30 min incubation with phenacetin (2–20 μM) in the absence or presence of CEP (0–50 μM). All data represent the mean of the incubations (performed in triplicate).

Lineweaver–Burk plots were employed to estimate the enzyme kinetic parameters for the probe reaction. The inhibitory kinetic data suggested that the inhibition of CYP3A4 was best fitted in a non-competitive manner (Figure 3(A)), and CYP2E1 (Figure 4(A)) and CYP2C9 (Figure 5(A)) was inhibited by CEP in a competitive manner. In addition, the *Ki* values of CEP on CYP3A4 (Figure 3(B)) CYP2E1 (Figure 4(B)) CYP2C9 (Figure 5(B)) were also obtained, with the values of 8.12, 11.78 and 13.06 μM, respectively.

### Time-dependent inhibition

**Figure 6.**
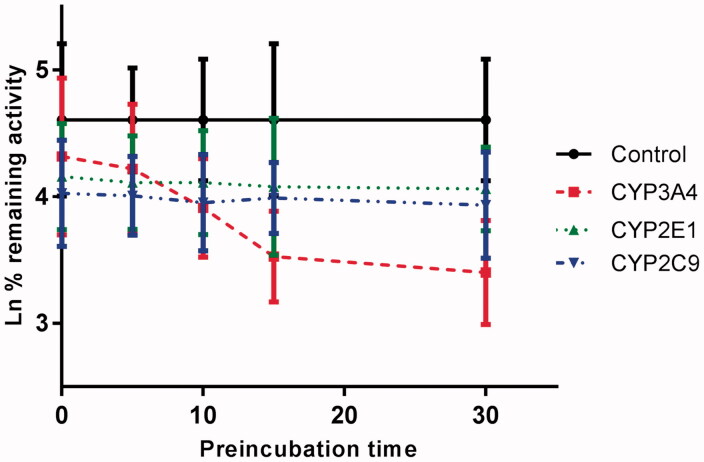
Time-dependent inhibition investigations of CYP3A4, CYP2E1, or CYP1A2 catalysed reactions by CEP (20 μM). All data represent the mean of the incubations (performed in triplicate).

**Figure 7.**
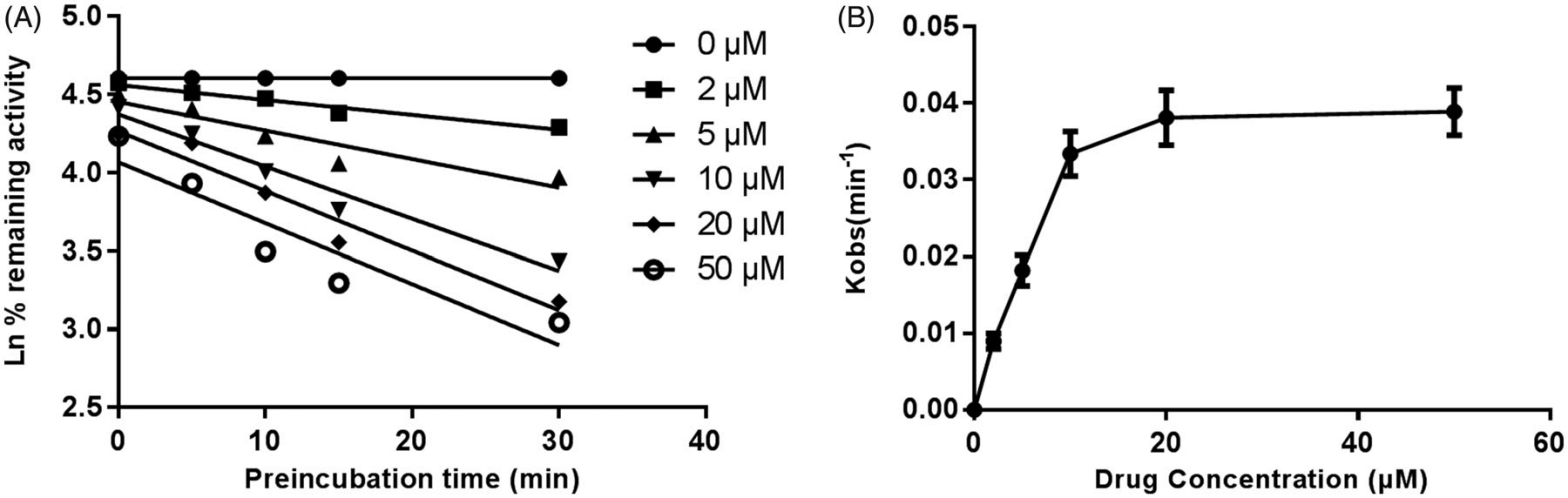
Time and concentration-inactivation of microsomal CYP3A4 activity by CEP in the presence of NADPH. The initial rate constant of inactivation of CYP3A4 by each concentration (*K_obs_*) was determined through linear regression analysis of the natural logarithm of the percentage of remaining activity *versus* pre-incubation time (A). The *K_I_* and *K_inact_* values were determined through non-linear analysis of the *K_obs_ versus* the CEP concentration (B).

Figure 6 shows the variation of CYP3A4, CYP2E1 and CYP2C9 with the incubation time, after pre-incubation of CEP with HLM for 30 min. The activity of CYP3A4 decreased with the incubation time, whereas, CYP2E1 and CYP2C9 were not affected. To characterize the time-dependent inhibition of CYP3A4 by CEP in HLM, the non-linear regression analysis was adopted to calculate the inactivation parameters of *K_I_* and *K_inact_* values. As calculated from the inactivation plot of Figure 7, the *K_I_*/*K_inact_* value for CYP3A4 was 10.84/0.058 min/μM. The *K_inact_* value indicated that about 5.8% CYP3A4 was inactivated each minute when a saturating concentration of CEP was incubated with HLM.

## Discussion

The activity of CYP enzymes plays a vital role in the metabolism of drugs. Nowadays, drug–drug interaction has become more and more popular, the modification of CYP enzymes should be paid more attention, especially the inhibition of CYP enzymes. Inhibition of CYP enzymes *in vivo* may result in unexpected elevations in the plasma concentrations of concomitant drugs, leading to adverse effects (Hu et al. 2015; Liu et al. 2015).

CEP has been widely used as a therapeutic agent (e.g., leucopoenia, hair loss) (Kometani et al. 1985), and reported to have effects of biomembrane stabilisation, improved peripheral circulation, inhibition of lipid peroxidation and anti-allergic action (Nakazawa et al. 1986; Furusawa and Wu 2007). Therefore, it is essential to investigate the effect of CEP on the activity of CYP enzymes. In this article, the effect of CEP on the metabolism of probe substrates of several CYP isoforms was investigated, including CYP1A2, CYP3A4, CYP2A6, CYP2E1, CYP2D6, CYP2C9, CYP2C19 and CYP2C8. The CYP3A subfamily is of high importance, as it is responsible for the metabolism of 60% xenobiotics (Basheer and Kerem 2015; Srinivas 2016). Among the subfamily, CYP3A4 is one of the most abundant enzymes accounting for about 30% of the total CYPs in the liver, and CYP3A4 is involved in the metabolism of 50% drugs. To identify the potential drug–drug interactions and herb–drug interactions in human, it is necessary to characterize the activity of CYP3A4 that involved in the metabolism of drugs. The result revealed that CEP was a weak inhibitor of CYP3A4, with the *Ki* of 8.12 μM and IC_50_ of 16.29 μM, which meant the potential of drug–drug interaction with CYP3A4 would also be low. On the other hand, the inhibitory effect of CEP was time-dependent, with the *Ki*/*K_inact_* value of 10.84/0.058 min/μM, which indicated that CEP would inhibit the activity of CYP3A4 with the increase of incubation time. From the results above, CEP should be used carefully when it combines with the drugs metabolized by CYP3A4 in clinic.

As previous studies reported, CYP2E1 and CYP2C9 play a vital role in the metabolism of many drugs (He et al. 2009; Lim et al. 2014; Xu et al. 2017; Bedada and Neerati 2018). There are also many drugs inhibiting the activity of CYP2E1 and CYP2C9 (Pan et al. 2010; Jiang et al. 2016; Liu et al. 2017; Hao et al. 2018), and we get similar results in the study of CEP. Different from CYP3A4, the activity of human liver microsomal CYP2E1 and CYP2C9 was inhibited by CEP competitively. Therefore, in order to avoid the adverse drug–drug interaction, CEP should be used carefully when co-administrated with the drugs metabolized by CYP2E1 and CYP2C9.

The above results are from the *in vitro* HLMs, which can provide the possibility for the understanding of the inhibition or DDI *in vivo*. However, the *in vitro* inhibition of CYP enzymes cannot ascertain the drug will cause clinically relevant interactions. Drug interaction mediated by CYP inhibition can be influenced by various factors, such as the contribution of the hepatic clearance during the metabolism of the drug, the fraction of the hepatic clearance which is subject to the inhibition of metabolism, and the ratio of the inhibition constant (*Ki*) over the *in vivo* concentration of the inhibitor (Ito et al. 1998; Ericsson et al. 2014). Therefore, further *in vivo* studies are essential for the interaction between CEP and CYP enzymes.

According to previous studies about the pharmacokinetic profiles of CEP in rats, the plasma *C*_max_ values of CEP in orally administered with CEP (10 mg/kg) were approximately 0.05 μg/mL (Jiang et al. 2016), which is much lower than the *Ki* and *IC_50_* values of CEP. Consequently, the drug–drug interaction might not occur when CEP was co-administered with the substrates of the CYP3A4, CYP2E1 and CYP2C9. Considering the pharmacokinetic differences of humans and rats, further *in vivo* system studies are needed to identify the interactions of CEP with CYP isoform in humans.

However, there are also some limitations to this study. We found the inhibitory effect of CEP on the activity of CYP3A4, 2E1 and 2C9 and the kinetic characteristics were also obtained. The potential mechanism requires additional investigation. Therefore, the inhibitory mechanism underlying effects of CEP on the activity of CYPs needs more attention. On the other hand, during the biotransformation of drugs, UDP-glucuronosyltransferases is also involved. In fact, the effect of CEP on the activity of UDP-glucuronosyltransferases had not been studied. In future research, the effects of CEP on the activity of UDP-glucuronosyltransferases should also be investigated *in vitro* and *in vivo*.

## Conclusions

The effect of CEP on the activity of CYP enzymes was investigated in this article. CEP inhibited the activity of CYP3A4, and competitively inhibited CYP2E1 and CYP2C9, while other CYP enzymes were not be influenced. Therefore, CEP should be used carefully together with the drugs metabolized by CYP3A4, CYP2E1 and CYP2C9.
